# Healthcare Pathways of Patients with Long COVID in Austria: A Qualitative Exploration of Experiences, Barriers, and Needs

**DOI:** 10.3390/jcm15114125

**Published:** 2026-05-27

**Authors:** Katharina Singer, Walter Struhal, Susanne Rabady

**Affiliations:** 1 Department of General and Family Medicine, Karl Landsteiner University of Health Sciences, Dr. Karl-Dorrek-Straße 30, 3500 Krems, Austria; katharinasinger@icloud.com (K.S.);; 2 Department of Neurology, University Hospital Tulln, Alter Ziegelweg 10, 3430 Tulln, Austria

**Keywords:** long COVID, post COVID, post-acute sequelae of COVID-19, Austrian healthcare system, qualitative research, healthcare pathways, patient experience

## Abstract

**Background/Objectives:** Long COVID is characterized by persistent symptoms following SARS-CoV-2 infection and places a considerable burden on patients and healthcare systems due to its complex, multisystemic nature. In Austria, little is known about how affected individuals navigate existing healthcare structures and where obstacles occur. This study aimed to explore healthcare pathways, perceived barriers, and needs among people living with long COVID in Lower Austria. **Methods:** An exploratory qualitative study was conducted using semi-structured interviews with eleven adults residing in Lower Austria who reported symptoms persisting for at least four months after COVID-19 infection and still present at interview. Participants were recruited from a rehabilitation center, a neurology department, and an online patient group. Interviews were audio-recorded, transcribed verbatim, pseudonymized, and analyzed by the first author using inductive qualitative content analysis following Mayring, supported by MAXQDA 2024 software. **Results:** On average, each participant consulted five medical points of care and seven healthcare professionals. Approximately half utilized Austria’s private healthcare sector in addition to the public one. Key barriers included fragmented care coordination, long waiting times, lack of specialist availability, financial burden, and insufficient recognition of symptoms by healthcare providers. Rehabilitation services were widely perceived as beneficial. **Conclusions:** Care experiences of the interviewed individuals with long COVID in Austria frequently deviate from national guideline recommendations. Although findings cannot be generalized beyond this exploratory sample, they suggest that enhancing general practitioner (GP) training, reinforcing care coordination, and broadening access to specialized interdisciplinary centers may improve equity and quality of long COVID care.

## 1. Introduction

Since the emergence of SARS-CoV-2 in early 2020, COVID-19 has affected millions of people worldwide [1]. While most patients recover within weeks, others experience symptoms that persist for months, impairing quality of life and daily functioning [2,3]. The World Health Organization defines post COVID-19 condition as symptoms occurring three months from the onset of COVID-19, lasting at least two months, and not attributable to alternative diagnoses [4]. The National Institute for Health and Care Excellence (NICE) categorizes the condition chronologically into different phases: an acute phase (signs and symptoms up to four weeks), an ongoing symptomatic phase (between four and 12 weeks), and a post-COVID syndrome (more than 12 weeks). Signs and symptoms lasting more than four weeks can also be referred to as long COVID syndrome [5]. In the literature and in clinical practice, the terms “long COVID” and “post-COVID/post COVID-19 condition” are often used interchangeably. For consistency and in line with current patient-facing and international usage, the term “long COVID” is used throughout this manuscript.

In general, long COVID can be classified as a post-viral syndrome. Existing scientific research on the pathogenesis of it remains incomplete, as the syndrome presents with multifactorial symptoms that are not uniform across all patients. Clinical presentation includes fatigue, dyspnea, exhaustion, cognitive impairment, insomnia, cardiovascular, and psychiatric manifestations [6,7].

In Austria, national guidelines like the Austrian S1 guideline for post-viral syndromes [7] and the “Aktionsplan zu postakuten Infektionssyndromen” (PAIS) [8] define primary care/general physicians (GPs) as the first point of contact and multidisciplinary coordinators of long COVID cases. The severity of symptoms will determine whether to opt for watchful waiting, referral for further specialist consultation or immediate medical intervention. Further diagnostics should only be performed if complaints persist for over 12 weeks, or red flags are present (for instance, clouding of consciousness, syncope, arrhythmia, signs of congestion or cyanosis). Referral to specialized long COVID clinics is recommended for complex cases and those not improving after one year of long COVID. Despite these frameworks, patient organizations and preliminary reports indicate that diagnostic processes are often prolonged, care is fragmented, and access to specialized services limited [9,10]. Interdisciplinary specialized clinics dedicated to addressing long COVID are planned to be established in Austria; however, nationwide coverage is currently not available.

International studies from the UK and the US report similar challenges: inconsistent symptom recognition, lengthy waiting times, and poor coordination between care levels [11,12,13]. Qualitative data capturing the real-world experiences of Austrian patients remain scarce; however, this is needed as the topic of underprovision is frequently discussed by the public.

In general, it seems necessary to clarify responsibilities, provide every player with specific assignments, use resources wisely and strive for a smooth collaboration of all disciplines that ultimately benefits the patient [10].

The aim of this study was to explore healthcare pathways, barriers, and perceived needs among people living with long COVID in Lower Austria, focusing on how care is accessed, coordinated, and experienced.

## 2. Materials and Methods

An exploratory qualitative study was conducted with eleven adults residing in Lower Austria who reported symptoms persisting ≥4 months after a COVID-19 infection between July 2022 and July 2023. Inclusion criteria were: (1) self-reported persistent symptoms following a confirmed or suspected SARS-CoV-2 infection lasting at least four months; (2) age at infection between 18 and 99 years; and (3) main residency in Lower Austria. All genders were included. A formal long COVID diagnosis was not a prerequisite at study entry; however, by the time of the interview all participants had carried symptoms for at least three months and therefore met the WHO definition of post COVID-19 condition [4]. Children were excluded because pediatric care in Austria is organized through separate structures. The four-month threshold was chosen to ensure that participants could reflect on a settled, persistent illness trajectory rather than on acute or early post-acute symptoms, and to align recruitment with the WHO definition of post COVID-19 condition [4]. Participants were recruited from a rehabilitation center, a neurology department, and an online patient group. The neurology department distributed approximately 120 recruitment posters to eligible patients, and the online patient group (Long COVID Austria) disseminated the poster through its digital channels twice. Interested individuals were encouraged to contact the first author themselves. At the rehabilitation center, eligible individuals who had previously consented to be approached for research (about 50–60 patients) were contacted by the first author by telephone. In total, one interview resulted from the neurology department, two from the online patient group, and eight from the rehabilitation center. Recruitment was conducted between autumn 2023 and autumn 2024. Despite an initial target of twenty interviews, only eleven individuals meeting the inclusion criteria agreed to participate. The cognitive and physical burden of long COVID, as well as the limited number of recruitment channels, likely contributed to this. Data saturation was not formally assessed, the exploratory aim of the study was to describe the diversity of care pathways rather than to achieve thematic saturation, which is acknowledged as a limitation. Potential participants received written study information, and the informed consent was obtained before interviews. The semi-structured telephone interviews were conducted, audio-recorded, transcribed verbatim and pseudonymized by the first author (K.S.).

Inductive qualitative content analysis following Mayring was applied using MAXQDA 2024, Berlin, Germany [14]. Three main categories were defined a priori based on the interview guide: (1) participant characteristics; (2) symptom development and management; and (3) healthcare system experience. Subcategories emerged through iterative line-by-line coding. Coding and categorization were refined through repeated transcript review. Coding followed an essentially inductive logic within these three pre-defined category domains and can therefore be characterized as a hybrid approach. All interviews were coded by the first author (K.S.), and category development, coding decisions, and emerging interpretations were discussed repeatedly with the senior supervisor (S.R.) to minimize individual interpretive bias. As no second independent coder was available, no formal inter-coder agreement statistic was calculated; instead, credibility was supported through iterative team discussions and the use of verbatim quotes to illustrate each theme. MAXQDA was used both for coding and to support category organization and retrieval of coded segments. The AI-assisted summarization function of the beta version was used only to support the exploration of coded segments and was verified manually. A reporting checklist based on the Consolidated Criteria for Reporting Qualitative Research (COREQ) was followed [15]. Researcher positionality: the first author (K.S.) was a medical student at Karl Landsteiner University and was not personally affected by long COVID; the second author (W.S.) is head of the consulted neurology department with clinical experience in post-viral syndromes; and the senior author (S.R.) is a general practitioner with research interests in long COVID care. The research team’s clinical and academic backgrounds inform an interpretive stance sympathetic to primary care, which was openly reflected upon during analysis.

The study was approved by the Ethics Committee of Karl Landsteiner University (protocol code No. 1028/2023, initial approval in September 2023, amendments approved in March and September 2024) and conducted in accordance with the Declaration of Helsinki [16]. Written informed consent, including permission to audio-record the interview and to use pseudonymized quotations, was obtained from all participants by postal return prior to the interview. Audio recordings and identifying information were stored on a password-protected, university-administered laptop accessible only to the first author and the senior supervisor. After transcription, personal identifiers (e.g., names, dates of birth) were replaced with participant codes, and audio files were deleted thereafter. All reported quotations are pseudonymized. Data handling complied with the EU General Data Protection Regulation (GDPR) and Austrian implementation legislation.

The research question addressed was: “What is the experience of patients who report to be suffering from post-acute sequelae after a COVID-19 infection regarding their care within the Austrian healthcare system?”

## 3. Results

A total of 11 participants were interviewed. The median age was 41 years (range 27–58). All were employed at the time of infection; 82% had medium or high occupational exposure risk (Table 1). Two reported pre-existing chronic health conditions.

Two main categories focus on the development of symptoms/management and healthcare system experience. Qualitative analysis guided the further subdivision into ten categories and 19 subcategories. A total of 472 segments were allocated to the development of symptoms and management, with an emphasis on the medical history, the development of symptoms, the management of long COVID, the current health status and the diagnoses made. The healthcare system experience was divided into 442 coded segments and five categories: initial medical contact, medical points of care, experience with the healthcare system, evaluation of alternative approaches, and requests and recommendations. The categorization is outlined in Table 2, with quotations provided to support it.

### 3.1. Development of Symptoms and Management

#### 3.1.1. Medical History

The participants’ COVID-19 infections took place between July 2022 and July 2023. All of them experienced symptoms within the first 4 weeks.

#### 3.1.2. Development of Symptoms

The average participant experienced five and a half symptom groups (e.g., neurological, respiratory, gastrointestinal) between 1 and 6 months and four symptom groups between 7 and 24 months. Five participants attributed their ongoing complaints to their SARS-CoV-2 infection right away, while six participants recognized the link only after a mean delay of approximately 38 days (1¼ months).

#### 3.1.3. Management of Long COVID

All participants had to take sick leave due to their long COVID symptoms, 45% were off work for several months. Three participants changed their job profile; however, no one was dismissed.

With regard to insurance-related concerns, four participants encountered challenges with Austrian health insurance providers. One participant verbalized: “I feel like a felon. I’ve been vaccinated three times and complied with all the lockdown regulations, and I always feel like a felon. Now I have to prove that I’m actually ill, that I’m not faking it and everyone in the system is constantly trying to somehow get me sent to a psychiatric ward.” Regarding prolonged sick leave, three participants experienced delays in the approval or rejection of sick leave claims, pressure to return to work prematurely, and the need to repeatedly submit medical documentation and proof of their sickness. 

Long COVID symptoms improved in six participants with the help of 1–4 factors/therapy options, while for five people five or more factors helped (overall mean of 4.2). Factors were, for example, dietary changes, warm temperatures, eye exercises or physiotherapy.

Two challenges which were stated by most (*n* = 8) of the participants were the psychosocial impact and the limited therapeutic options, followed by the financial burden (*n* = 6). All of the interviewees found themselves paying for a service or product that was not covered by their health insurance company.

Seven of the people interviewed (*n* = 7) said that specialists were (at least once) difficult to find.

#### 3.1.4. Current Health Status

All of the participants were still experiencing long COVID symptoms at the time of the interview. The most prevalent symptoms reported were exhaustion (*n* = 7), followed by neurological issues (like chronic fatigue and/or headache) and neuropsychological complaints (like insomnia and/or cognitive dysfunction) (*n* = 6).

#### 3.1.5. Diagnoses

All participants have a long COVID diagnosis. Five participants attributed their ongoing complaints to their SARS-CoV-2 infection right away, leading to a diagnosis at 3 months (approximately 90 days) after initial infection, when the diagnostic criteria were met. Six participants recognized the link only after a mean delay of approximately 38 days (1¼ months) and were diagnosed at around 128 days (4¼ months; range 4–7 months) post-infection.

The median number of diagnoses was two per participant (mean 1.7). The general practitioner was involved in the diagnostic process for each participant (primary point of contact). On average, four specialties of medical professionals were involved in the diagnostic process (mean 3.7, standard deviation (SD) 1.62). Participants who reported that their diagnosis had been made per exclusion (*n* = 5) were involved with five specialties (mean 5.2, SD 0.84).

### 3.2. Healthcare System Experience

#### 3.2.1. Initial Medical Contact

**Figure 1:**
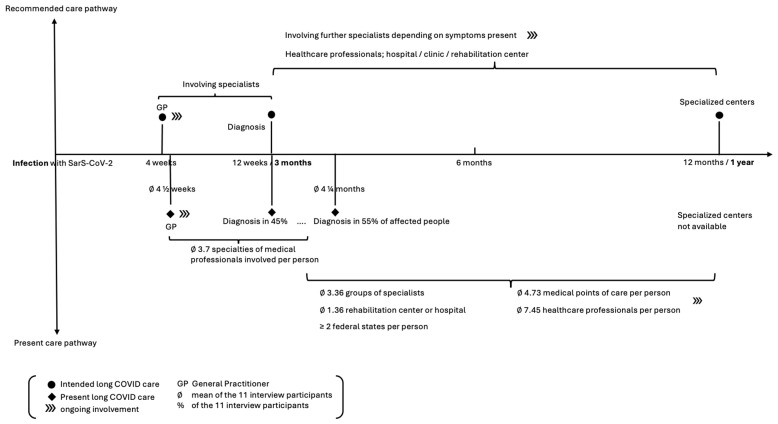
Long COVID care pathways. Schematic representation of the recommended healthcare pathway presented above the *x*-axis/timeline, and the present care pathway experienced by participants with long COVID in Lower Austria based on the interview data is presented below it.

The first point of contact for all 11 interview participants with SARS-CoV-2 related symptoms was their general practitioner. Seven patients contacted their GP with long COVID complaints, while the others (*n* = 4) visited their GP earlier. On average, the first medical contact was made after approximately 32 days (4½ weeks). Approximately half of the GPs referred their patients (*n* = 5, 45%) to specialists, while the remaining (*n* = 6) opted for a wait-and-see approach, and some (*n* = 4) prescribed symptomatic therapy. The further diagnostic pathways were non-linear, depending on the symptoms present. A schematic overview of the observed care pathways, from the general practitioner as first contact to subsequent specialist, rehabilitation, and hospital-based services, is shown in Figure 1.

#### 3.2.2. Medical Points of Care

On average, each patient contacted specialists of three medical disciplines (mean 3.36), for instance one general practitioner and pulmonologist and neurologist. Also, each patient visited one rehabilitation center or hospital (mean 1.36, SD 0.67). That makes a total of approximately five medical points of care (mean 4.73) per person. When counting each healthcare professional visited, not by number of visits, but by individual person or facility, the average study participant visited/involved seven (mean 7.45, SD 3.45) healthcare professionals.

All of the participants had their main residency in Lower Austria. In terms of the locations where medical care was sought, a total of 82 healthcare professionals were consulted across all participants. The location was known for 49 of these professionals, representing 60% of the total. Of these 49 identified locations, 29 (59%) were located in Lower Austria, 19 (39%) in Vienna, and one (2%) in Burgenland. Notably, all interviewees sought medical care in both Lower Austria and Vienna.

Two of the participants had private insurance coverage. Additionally, three participants utilized private healthcare services. So, approximately half of the participants utilized Austria’s private healthcare system in addition to the public one, while the remaining participants exclusively accessed the public healthcare sector.

All primary care physicians, also referred to as the general practitioners, were located in Lower Austria. Four interviewees reported positive experiences with their GPs, five highlighted early referrals to specialists and three mentioned efforts to educate themselves about long COVID. Another four interviewees reported ambivalent experiences, reporting complications with securing timely appointments and diagnostics. Three interviewees presented negative experiences with their GPs, outlining primarily a perceived lack of validation of their symptoms. Some (*n* = 4) tried to find another GP in their area. Of these, one patient was successful in locating a new GP, while two interview participants were unsuccessful and one contacted a private GP.

In addition to the general practitioner, the study participants also visited other healthcare professionals. The contacted pulmonologists (eight study participants) conducted tests such as lung function tests and CT scans. Five participants expressed satisfaction with the care they received, noting that the pulmonologists took the time to explain their conditions and provide appropriate treatments, including referrals for pulmonary rehabilitation. The other three expressed dissatisfaction, reporting dismissive pulmonologists that were unable to provide a diagnosis or treatment plan. Of the eight participants who visited a pulmonologist, three were in Vienna, four in Lower Austria and one in Burgenland. The participants found the pulmonologists either through personal connections, based on recommendations from their GPs/colleagues or via the internet. 

Five patients (*n* = 5) sought neurological care. These healthcare professionals diagnosed two of the participants (*n* = 2) with mast cell activation syndrome and two (*n* = 2) with chronic fatigue syndrome. Two participants expressed struggles in accessing neurological long COVID care. 

Cardiologists were consulted by four interviewees (*n* = 4). Three of these visits (*n* = 3) did not result in effective treatment.

In addition, ear, nose and throat doctors (*n* = 2), psychotherapists (*n* = 2), physiotherapists (*n* = 2), a gastroenterologist (*n* = 1) and specialists for Japanese (*n* = 1) and Chinese medicine (*n* = 1) were consulted.

The majority of participants (*n* = 10) attended a rehabilitation program (notably, eight of the study participants were recruited via one of these rehabilitation centers). These respective ten participants visited, on average, one rehab center (mean 1.3, SD 0.67). Program formats ranged from short-term inpatient to long-term outpatient rehabilitation. Participants attended between one and three cycles of rehab. The programs were generally perceived as beneficial by all ten of these participants, particularly with regard to the acquisition of new strategies for coping with long COVID. Six participants highlighted the up-to-date knowledge of the healthcare professionals involved and two participants appreciated the timely initiation of the programs. Psychosocial support, mindfulness exercises, breathing techniques, and pacing strategies, as well as physical activities and (training) plans for daily life, were mentioned positively. Negative aspects included too limited time for group-talk sessions (*n* = 2) and deterioration of symptoms due to overly activating therapies (*n* = 2). 

Five participants sought help from hospitals and visited, on average, two hospitals (mean 1.8, SD 0.84). In total, nine visits were made, of which five were classified as inpatient visits and four as outpatient visits. The hospitals were perceived as supportive based on their acknowledgement of symptoms and provision of referrals. In one case, a participant positively highlighted the interdisciplinary collaboration. In contrast, one participant was rejected due to lack of availability. Another participant received a standardized consultation consisting of a predetermined series of questions, which she perceived as not aligning with her symptoms or medical history.

#### 3.2.3. Experience with the Healthcare System

The interview participants all reported positive as well as negative experiences with the Austrian healthcare system. Categories were identified to classify the experiences most frequently mentioned.

In terms of care coordination, six participants reported lacking a consistent/responsible point of contact, five participants would have wished for a referral they did not get, and four participants faced organizational/bureaucratic difficulties. In terms of patient-centered care, six participants felt that their condition was not taken seriously/misjudged, five participants felt a lack of support and four felt that they were not listened to.

The average positive and negative experiences per participant were grouped into two categories: those who accessed only the public healthcare sector (*n* = 6) and those who accessed both the public and private sectors (*n* = 5). For the group who accessed only the public healthcare sector, there was a higher number of categories mentioned positively and a lower number of categories mentioned negatively. 

The comparison of positive and negative experience showed the greatest dissatisfaction in the categories “care coordination” (*n* = 10) and “patient-centered care” (*n* = 11). “Public awareness” was experienced positively by two participants. 

The limited availability of long COVID clinics was a recurring theme.

In summary, the most frequently perceived barriers included:

1. Fragmented care—lack of central coordination, inconsistent referral patterns.
2. Access limitations—long waiting times, shortage of specialists.
3. Financial burden—out-of-pocket expenses up to €600/month for non-reimbursed therapies and supplements.
4. Lack of recognition &/validation—skepticism from insurance physicians, misdiagnoses.
5. Insufficient therapeutic options—no causal treatment available.
6. Psychosocial strain—isolation, frustration, loss of self-identity.

#### 3.2.4. Evaluation of Alternative Approaches

Slightly more than half of interviewees reported that, in hindsight, they would go for alternative approaches and would address some of the situations experienced in a different manner. Seven participants expressed that they would have sought medical attention earlier, either by contacting emergency services, requesting referrals to specialists or pursuing private healthcare options. Two respondents also mentioned that they would seek a second opinion and look more closely for healthcare professionals who were specialized in post-viral syndromes/long COVID. Four participants emphasized the need for quicker access to rehabilitation services.

#### 3.2.5. Requests and Recommendations

Regarding requests and recommendations for future improvement, the people affected by post-acute sequelae of COVID-19 (PASC) provided an average of five requests (mean 5.0, range 3–7).

Participants most frequently suggested (*n* = 11, multiple mentions per participant possible):

- Care Coordination—Predefined pathways for patient care; improved coordination and quicker referral. GP as the central figure for case management. (*n* = 9)
- Physician Training—Mandatory and continuous education for healthcare providers on long COVID and related conditions. (*n* = 9)
- Patient-Centered Care—Personalized care plans based on individual needs and experiences; active listening by providers. (*n* = 8)
- Regular Updates—Repeated communication/consult regarding diagnosis, treatment options and the latest research. (*n* = 8)
- Access to Therapies—Broader (insurance-covered) access to therapy. (*n* = 6)
- Financial Support—Accessible financial and bureaucratic support, disability benefits and coverage for medical expenses. (*n* = 6)
- Specialized Clinics—The establishment of designated centers for post-viral conditions offering multidisciplinary care. (*n* = 5)
- Public Awareness—Increased education for the public on the impact and legitimacy of long COVID. (*n* = 4)

## 4. Discussion

### 4.1. Development of Symptoms and Management

The present study illustrates that participants frequently experience prolonged uncertainty regarding the nature of their symptoms. Although all reported complaints occurred within the first four weeks, many (55%) only suspected a connection to their SARS-CoV-2 infection retrospectively. The diagnosis of long COVID was made between three and seven months after the initial infection. This mirrors current evidence that symptoms evolve slowly and inconsistently, delaying recognition and making early diagnostic clarity difficult [4]. The prevalence of fatigue, cognitive dysfunction, sleep disturbances, and exertion-related deterioration aligns with international data describing long-term multisystem involvement [2].

Sick leave and work adaptations were common, yet none of the participants lost their employment, which contrasts with reports from other countries of work instability among long COVID patients [18]. The professional backgrounds of this cohort, with many members employed in public service sectors, may offer a partial explanation for this observed difference. Nonetheless, participants described substantial psychosocial strain, including loss of autonomy, and fear/frustration of not being believed. One third of the participants reported significant challenges related to insurance and administrative matters. A recent report emphasized the necessity for bureaucratic assistance, particularly in relation to insurance-related matters [19]. Financial burden was another central theme: all interviewees paid privately for therapies or services not covered by insurance. One participant accumulated more than €16,000 in costs for diagnostic and therapeutic attempts. These findings underscore the substantial socioeconomic weight of long COVID, consistent with recent studies documenting high personal spending, even in the absence of clear therapeutic benefit [13,20].

Participants relied on a wide range of symptom-management strategies. While improvements were noted in some cases, particularly through changes in diet, warm temperatures, breathing therapy, or physiotherapy, many described slow progress and limitations. These observations are in line with international data showing that multimodal, symptom-led strategies (including pacing, graded activity, and respiratory rehabilitation) currently form the backbone of long COVID management in the absence of a causal therapy [10,11]. The emphasis on self-management aligns with national guideline recommendations and reflects the scarcity of targeted treatment options [7].

Diagnostic journeys were often complex and non-linear. On average, four medical specialties and seven healthcare professionals were involved; however, one patient contacted/involved 16 healthcare professionals. Approximately half of the cohort received a diagnosis soon after meeting the established criteria (definition of long COVID: persisting symptoms 3 months after SARS-CoV-2 infection), while the remainder required a longer period of time (approximately 128 days, i.e., 4¼ months). This variability suggests differences in provider awareness, diagnostic confidence, and access to appropriate evaluations. Similar heterogeneity in diagnostic timelines has been described in US [11] and UK [13] cohorts, and recent work from Sweden has highlighted how ongoing epistemological uncertainty around long COVID-19 condition translates into inconsistent clinical recognition [21]. The participants reported that the absence of objective biomarkers and the inconsistent knowledge of healthcare providers regarding long COVID contributed to repeated assessments, referrals, and uncertainty.

### 4.2. Healthcare System Experience

General practitioners served as the first point of contact for all participants, confirming their central role as suggested in national and international recommendations. Experiences, however, differed markedly. Some participants reported attentive GPs who referred them early to specialists or sought additional knowledge about long COVID, while others experienced difficulties obtaining adequate referrals or follow-ups and perceived a lack of seriousness. Several participants attempted to change their GP, but not all succeeded, highlighting workforce and access constraints in primary care. The constraints imposed by limited consultation time and inadequate reimbursement were further identified as barriers to effective management by study participants and the international literature [11].

Participants repeatedly emphasized the difficulty of locating clinicians who are knowledgeable about long COVID, even in urban areas, alongside with long waiting times. The absence of specialized long COVID clinics or reference centers in Lower Austria resulted in a significant number of participants seeking care in Vienna. The rehabilitation programs were attended by almost all participants and were widely perceived as positive and validating. Rehabilitation measures, tailored to the presenting symptoms, are also strongly recommended by the Austrian S1 guideline [7].

Deficits in care coordination were a dominant theme: many participants had no consistent point of contact, received limited guidance through diagnostics, had to arrange appointments themselves, and sometimes did not obtain requested referrals. These experiences align with prior Austrian analyses of fragmented pathways and unclear responsibilities, and the 2025 launch of a private “Long COVID Online-Klinik” further signals unmet demand for specialized services [10,22,23]. Comparable gaps are reported in the UK and US, where multidisciplinary clinics are associated with higher patient satisfaction and clearer care pathways [11,12,13]. Within Europe, Austria, Germany, and Switzerland face broadly comparable structural challenges but differ in their responses: Germany has moved to dedicated reimbursement codes and a centralized information portal and has formally recognized long COVID as an occupational disease in defined settings, while Switzerland has prioritized research infrastructure and disability benefit pathways [10]. Austria’s Action Plan for Post-Acute Infection Syndromes (PAIS) adopts several of these elements conceptually, including transdisciplinary contact points, a national reference center, and adapted social security provisions, yet implementation remains uneven [8,10]. Our findings suggest that, at the policy level, three levers appear particularly relevant: (1) appropriate reimbursement for time-intensive GP consultations and structured assessment (e.g., red-flag screening, post-exertional malaise screening, orthostatic testing), which would allow primary care to take on the coordinating role envisaged in the guidelines [7,11]; (2) binding referral pathways linking primary care to regional interdisciplinary long COVID centers, so that patients do not rely on personal initiative or socioeconomic resources to access specialist input; and (3) strengthened social-security frameworks (sick leave, rehabilitation, and return-to-work support), addressing the bureaucratic burden reported here and elsewhere [19]. Conceptually, our findings map onto three widely used frameworks in health-services research: “care continuity” (informational, management, and relational continuity were all reported as lacking), “health-system navigation” (participants had to identify and sequence providers themselves, often without a guiding professional), and “patient-centered care” (active listening, validation, and shared decision-making were the attributes most frequently missed). Aligning Austrian implementation with these frameworks may help translate existing guideline content into routine practice.

To conclude, the weaknesses of the health care system identified by patients align closely with the international literature [11,12,13,18,21].

### 4.3. Strengths and Limitations

A key strength is the inclusion of patients recruited from different entry points, enabling diverse perspectives. The focus on Lower Austria allows for a coherent in-context analysis, since the availability of long COVID care varies substantially between Austrian federal states. The use of a semi-structured interview guide informed by a prior literature review, supported by COREQ-aligned reporting [15], strengthens methodological transparency.

Limitations include the restriction to one federal state (Lower Austria) and the small sample size of eleven participants. Several factors should be considered when interpreting the findings. First, eight of the eleven participants were recruited through a single rehabilitation center. Individuals accepted into a rehabilitation program may differ systematically from other long COVID patients, for instance by having more severe or medically recognized symptoms, better access to specialist referral, or more favorable views of rehabilitation services. This likely introduces a selection bias and may underrepresent those unable to access rehabilitation. Second, recruitment through a neurology department and an online patient group may additionally have skewed the sample towards patients with neurological or cognitive symptoms and towards more digitally engaged or activated individuals. Third, data saturation was not formally assessed: the study was designed as an exploratory investigation of care pathways, and findings should therefore be interpreted as illustrative of the range of experiences rather than as representative of all long COVID patients in Austria. Fourth, the majority of participants were employed in public service or similar stable occupations, which may explain the absence of dismissals and may limit transferability to self-employed individuals, those in precarious employment, or those working in the private sector, where financial and employment consequences may be more pronounced [18]. Fifth, all interviews were coded by a single researcher (K.S.) with ongoing supervision by the senior author. While this is acceptable in exploratory qualitative work and credibility was supported through iterative team discussions, the absence of a formal inter-coder agreement procedure should be acknowledged. Sixth, the study relied on self-reported SARS-CoV-2 infection and self-reported symptoms, without medical-record verification. Finally, recruitment was more difficult than anticipated; plausible reasons include the cognitive and physical burden of long COVID itself (several contacted individuals declined because of fatigue and cognitive disturbance), the requirement to contact the investigator actively, and the limited number of channels used.

## 5. Conclusions

This study indicates that the care experiences of individuals with long COVID in Austria frequently deviate from national guideline recommendations. While all participants initially sought care from their general practitioner, subsequent pathways were often fragmented, diagnostic work-up was at times delayed, and access to treatment proved inconsistent, shaped by geographic location, insurance status, and personal initiative. Rehabilitation services and supportive healthcare professionals emerged as key facilitators of care. Enhancing GP training and reinforcing their coordinating role, providing appropriate reimbursement for time-intensive consultations, and broadening access to specialized, interdisciplinary reference centers may help address current shortcomings and improve equity. Overall, more consistent implementation of existing guidelines appears necessary and likely to strengthen the quality of long COVID care.

## Figures and Tables

**Table 1:** Demographic and occupational characteristics of participants. Occupational exposure risk was categorized following the classification of the UK Environmental Modelling Group (EMG) report on COVID-19 risk by occupation and workplace [17]: high risk refers to occupations with frequent close contact to infectious individuals (e.g., healthcare workers, police officers on duty); medium risk to occupations with moderate public or colleague contact (e.g., teachers, public administration); low risk to occupations with limited in-person contact or predominantly remote work (e.g., legal professions, office-based remote work).

Variable	Category	*n* (%)
All Participants		*n* = 11 (100%)
Gender	Female	7 (64)
	Male	4 (36)
Age	18–30 years	2 (18)
	31–45 years	6 (55)
	≥46 years	3 (27)
Place of residence	Lower Austria	11 (100)
Occupational exposure risk [17]	High	3 (27)
	Medium	6 (55)
	Low	2 (18)

**Table 2:** Main categories, categories and subcategories with illustrative quotations.

Main Category	Category	Subcategory
Development of symptoms and management	Medical history “With symptoms of a cold, a flu-like infection, but which felt different than usual.”	Pre-existing health condition
		Symptoms (≤4 weeks)
		Previous COVID/similar complaints
	Development of symptoms “This severe feeling of illness didn’t actually start until four weeks later, and then didn’t go away.”	Recognition of long COVID
		Symptoms (>4 weeks)
	Management of long COVID “I do everything on the edge of my health and financial resources.”	Sick leave and insurance matters
		Improvement of symptoms
		Efforts to manage symptoms
		Sources of help received
		Additional challenges
	Current health status “It’s as if you are locked up.”	
	Diagnoses “I: But does that mean you now have fatigue or long COVID? 03: That’s a good question.”	
Healthcare System Experience	Initial medical contact “I think it was a good idea to get to the GP as quickly as I did.”	
	Medical points of care “He [neurologist] has not accepted any new patients for two years. He is fully booked.”	Primary care physician
		Pulmonologist
		Neurologist
		Cardiologist
		Other medical specialists
		Rehabilitation
		Hospital
	Experience with the healthcare system “I don’t know what else to do. Yes, I’m frustrated.”	Negative
		Positive
	Evaluation of alternative approaches “If I could go back, I would do things differently.”	
	Requests and recommendations “You just have to train doctors. I don’t know if you can make doctors train. I would just do it.”	

## Data Availability

The data presented in this study are available on request from the first author due to privacy and ethical restrictions (participant confidentiality).

## Abbreviations

The following abbreviations are used in this manuscript:

SARS-CoV-2	Severe Acute Respiratory Syndrome Coronavirus 2
COVID-19	Coronavirus Disease 2019
GP(s)	General Practitioner(s)
PAIS	Action Plan for Post-Acute Infection Syndromes (Aktionsplan zu postakuten Infektionssyndromen)
SD	Standard Deviation
CT	Computed Tomography
LCS	Long COVID Syndrome
PASC	Post-Acute Sequelae of COVID-19
UK	United Kingdom
US	United States
BMSGPK	Bundesministerium für Soziales, Gesundheit, Pflege und Konsumentenschutz
NICE	National Institute for Health and Care Excellence
SIGN	Scottish Intercollegiate Guidelines Network
JAMA	Journal of the American Medical Association
WHO EMG	World Health Organization Environmental Modelling Group
